# Identification of Small-Molecule Antagonists Targeting the Growth Hormone Releasing Hormone Receptor (GHRHR)

**DOI:** 10.1021/acs.jcim.4c00577

**Published:** 2024-08-29

**Authors:** Minos-Timotheos Matsoukas, Tarryn Radomsky, Vasilis Panagiotopoulos, Robin du Preez, Michail Papadourakis, Konstantinos Tsianakas, Robert P Millar, Ross C Anderson, Georgios A Spyroulias, Claire L Newton

**Affiliations:** † University of West Attica, Department of Biomedical Engineering, Athens 12243, Greece; ‡ Centre for Neuroendocrinology, Department of Immunology, Faculty of Health Sciences, University of Pretoria, Private Bag X323, Gezina, Pretoria 0031, South Africa; § Department of Physiology, Faculty of Health Sciences, University of Pretoria, Private Bag X323, Gezina, Pretoria 0031, South Africa; ∥ Cloudpharm PC, Athens 15125, Greece; ⊥ Deanery of Biomedical Sciences, University of Edinburgh, Edinburgh EH8 9JZ, U.K.; # Institute of Infectious Diseases and Molecular Medicine, Faculty of Health Sciences, University of Cape Town, Cape Town 7925, South Africa; ∇ School of Medicine, University of St Andrews, St Andrews KY16 9TF, U.K.; ○ University of Patras, School of Health Sciences, Department of Pharmacy, University Campus, Rion, Patras 26500, Greece

## Abstract

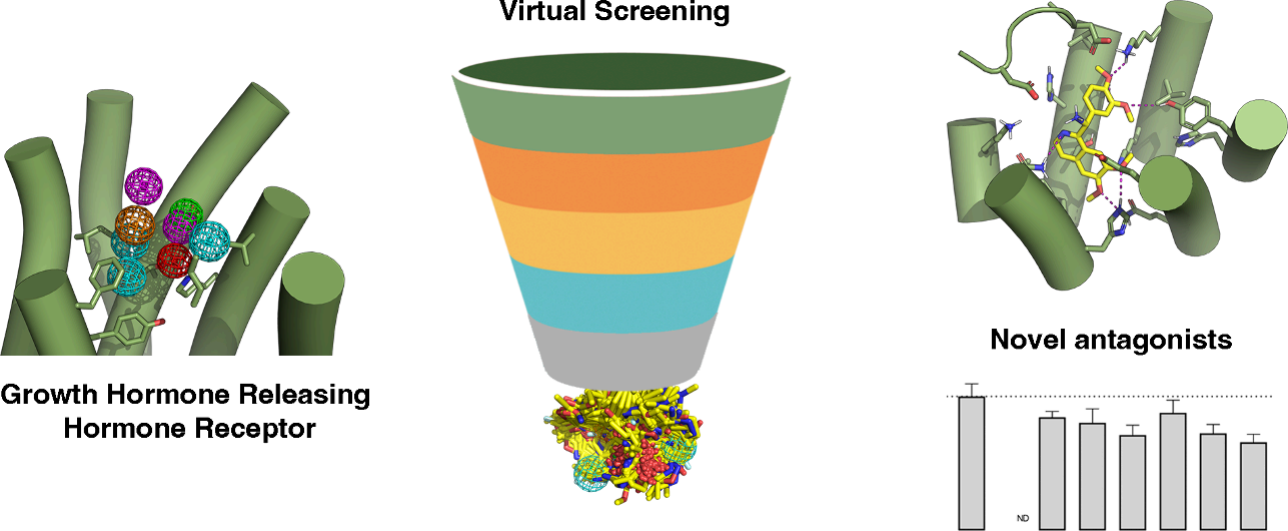

The growth hormone-releasing hormone receptor (GHRHR) belongs to Class B1 of G protein-coupled receptors (GPCRs). Class B1 GPCR peptides such, as growth hormone-releasing hormone (GHRH), have been proposed to bind in a two-step model, where first the C-terminal region of the peptide interacts with the extracellular domain of the receptor and, subsequently, the N-terminus interacts with the seven transmembrane domain of the receptor, resulting in activation. The GHRHR has recently been highlighted as a promising drug target toward several types of cancer and has been shown to be overexpressed in prostate, breast, pancreatic, and ovarian cancer. Indeed, peptide GHRHR antagonists have displayed promising results in many cancer models. However, no nonpeptide GHRHR-targeting compounds have yet been identified. We have utilized several computational tools to target GHRHR and identify potential small-molecule compounds directed at this receptor. These compounds were validated *in vitro* using a cyclic adenosine monophosphate (cAMP) ELISA to measure activity at the GHRHR. *In vitro* results suggest that several of the novel small-molecule compounds could inhibit GHRH-induced cAMP accumulation. Preliminary analysis of the specificity/selectivity of one of the most effective hit compounds indicated that the effect seen was via inhibition of the GHRHR. We therefore report the first nonpeptide antagonists of GHRHR and propose a structural basis for inhibition induced by the compounds, which may assist in the future design of lead GHRHR compounds for treating disorders attributed to dysregulated/aberrant GHRHR signaling.

## Introduction

G protein-coupled receptors (GPCRs) comprise the largest family of proteins in humans, which consists of more than 800 receptors.^1^ They are membrane proteins that are composed of seven transmembrane helices (TM1-TM7) connected by three extracellular (ECL1-ECL3) and three intracellular (ICL1-ICL3) loops (collectively termed the seven transmembrane domain; 7TMD).^2^ Class B1 (also known as Secretin-like) GPCRs possess a large structured extracellular N-terminal domain (ECD) and bind large peptides that are implicated in many physiological and pathophysiological conditions.^3^ Binding of the endogenous peptide ligands is believed to be a two-step process whereby the C-terminus of the peptide interacts with the receptor ECD, followed by interaction of the N-terminus of the peptide with the receptor 7TMD. These interactions are associated with conformational changes that are transmitted through the 7TMD to the receptors’ cytoplasmic region.^4^ Such structural changes are responsible for interaction of the receptors with, and activation of, G proteins that subsequently elicit multiple intracellular responses.^5^ Recent cryogenic electron microscopy (cryo-EM) and crystallographic structural findings for Class B1 GPCRs have rendered them more accessible targets in pharmaceutical research, assisting the design and synthesis of many targeted peptide and nonpeptide analogues.^6^ In recent years, advances in GPCR structural biology methods have enabled the characterization of several Class B1 GPCRs in complex with their peptide ligands and/or G proteins and have provided information about the structural mechanisms of ligand recognition and selectivity.^3,7^

The growth hormone-releasing hormone receptor (GHRHR), a Class B1 GPCR, is activated by growth hormone-releasing hormone (GHRH), a 44-residue neuropeptide produced in the hypothalamus that regulates the production and secretion of growth hormone (GH) by pituitary somatotropes. In turn, GH stimulates the secretion of hepatic insulin-like growth factor I (IGF-I), both of which are important for the regulation of growth and metabolism. Both GH and IGF-1 have also been found to act as potent mitogens in cancers.^8^ However, the role of these hormones in cancer remains to be fully elucidated and exploited in a therapeutic context, with GH and IGF-1 targeting strategies resulting in side effects such as fluid retention, hypertension, and increased body weight.^9^ Overexpression of GHRHR and its most prominent splice variant, SV1, has also been associated with several types of cancer, including breast,^10^ prostate,^11^ thyroid,^12^ pancreatic,^13^ and esophageal^14^ cancers. SV1, in which the first three exons of GHRHR are replaced with a fragment of intron 3, inducing a novel in-frame start codon, is known to have ligand-independent activity and is believed to confer growth-stimulating effects in many tumor tissues.^15^ GHRHR peptide antagonists (which block GHRH-induced and/or ligand-independent receptor activity) have been shown to inhibit growth of various cancer cell lines in addition to showing benefits in models of prostate,^16^ breast,^17^ pancreatic,^18^ gastric,^19^ and ovarian^7,20^ cancer. However, features of peptide antagonists, such as the need to inject, poor pharmacokinetic profiles, and issues regarding preparation synthesis/storage and uniformity, compromise the application of these peptides in a clinical setting. To date, no nonpeptide (small-molecule) compounds targeting the GHRHR have been reported. Thus, the identification of small-molecule antagonistic compounds targeting the receptor would have a possible clinical application in the treatment of several conditions, including cancer.

We have previously performed computational studies to propose structural elements related to relative ECD movement and activation of the GHRHR^21^ and, recently, cryo-EM structures of both the GHRHR and SV1 splice variant in complex with GHRH and the heterotrimeric Gαs (Gs) G protein have been determined.^7,22^ The application of cryo-EM to determine the structure of many GPCRs has provided a wealth of information to aid in the understanding of drug-receptor interactions. Furthermore, this has enabled the construction of increasingly accurate *in silico* homology models,^23,24^ also aided by recent advances in protein structure prediction with Alphafold,^24^ and has resulted in many successful small-molecule compound identification projects.^25^ Purchasable “fragment-like” or “lead-like” compound databases have enriched the available pool of small molecules for *in silico* virtual screening methodologies and subsequent *in vitro* testing. Indeed, several million organic molecules are commercially available, via services such as the ZINC (or other) databases.^26^ When initial hits toward a target are identified, they are usually used as templates in hit-to-lead optimization schemes through organic chemistry synthesis. Currently, the availability of commercially ready-to-test compounds facilitates a process called “optimization by catalogue” to rapidly explore the chemical space of an active hit without the need of actual chemical synthesis in the hit optimization process.^27^

In this work, we have constructed a receptor-based pharmacophore using a model of the GHRHR 7TMD and performed virtual screening on a commercially available library of ∼9 million compounds for the identification of small molecules targeting this interface. The virtual screening of the compound library yielded forty-four compounds to take forward into *in vitro* signaling assays. Cyclic adenosine monophosphate (cAMP) enzyme-linked immunosorbent assays (ELISAs) were performed, and a potential inhibitor was identified. This was followed up by exploring its chemical space through compounds within the virtual screening (VS) library to identify compounds with chemical similarity. These were then examined in the cell-based model to establish their ability to modulate GHRHR activity, and three were found to inhibit receptor activity. We therefore report a series of novel compounds that could serve as lead compounds for further refinement with the goal of developing GHRHR small-molecule analogues for use to examine the role of GHRHR in various pathologies in a preclinical setting, in addition to clinical application.

## Materials and Methods

### Materials

pcDNA3mammalian expression vector encoding the human GHRHR (NM_000823.4) with a C-terminal FLAG epitope tag was a kind gift from Prof Kelly Mayo (Northwestern University, USA). pcDNA3.1(+) mammalian expression vector encoding the human GLP-1 receptor (GLP-1R; NM_002062.5) was a kind gift from Prof Jae Young Seong (Korea University, South Korea). Empty vector (pcDNA3.1(+)) was purchased from Invitrogen (Carlsbad, CA, USA). GHRH (GHRH(1–29); Sermorelin) and GLP-1 were purchased from EZBiolabs (Carmel, IN, USA). The peptide antagonist, JV-1–36, was purchased from Phoenix Pharmaceuticals (Burlingame, CA, USA). Forskolin (FSK) was purchased from Sigma-Aldrich (St Louis, MO, USA). Small-molecule test compounds were purchased from Molport (Riga, Latvia).

### Homology Modeling

Modeler v9.17^28^ was used to build a homology model of the human GHRHR 7TMD in the inactive state (Uniprot ID Q02643). The structural model was built using the cryo-EM structure of the human glucagon receptor (GCGR) 7TMD domain (PDB code 4L6R) as a template.^29^ The conserved S152^1.50b^ in TM1, H177^2.50b^ in TM2, E245^3.50b^ in TM3, W272^4.50b^ in TM4, N318^5.50b^ in TM5, G359^6.50b^ in TM6, and G393^7.50b^ in TM7, were used as reference points in the 7TMD sequence alignment. For GHRHR, corresponding residues are S140^1.50b^, H165^2.50b^, E223^3.50b^, W250^4.50b^, N296^5.50b^, G339^6.50b^, and G369^7.50b^. Superscripts denote the single most conserved residue in each TM among Class B1 GPCRs, which is designated as X.50b (where X refers to the corresponding TM).^30^ The overall stereochemical quality of the homology model was evaluated by the discrete optimized energy (DOPE)^31^ and a thorough visual inspection and subsequently subjected to a 500-step energy minimization using the Amber 99SB-ILDN^32^ force field.

### Virtual Screening

The ∼9 million purchasable compounds of the Clean Drug-like subset of the ZINC database^33^ were filtered according to molecular weight (230–500 Da), AlogP (0–6), hydrogen bond acceptors (≥2), and aromatic rings (≥1) using Openbabel,^34^ finally leading to ∼2.6 million unique compounds. For each compound, 250 conformations were generated with a relative energy difference window of 20 kcal/mol.

The structural model of the GHRHR 7TMD was used to generate a pharmacophore model based on the chemical features of the orthosteric pocket residues, using the Discovery Studio 3.5 software.^35^ Eight pharmacophore features were manually added, mapping potential ligand interactions with residues Y133^1.43b^, K175^2.60b^, V179^2.64b^, S209^3.36b^, T213^3.40b^, D274^ECL2+2^, I285^5.39b^, Y342^6.53b^, F345^6.56b^, and L362^7.43b^. Thirty-six different pharmacophores, containing all combinations of seven out of eight features, were screened one at a time, as described previously.^36^ Excluded features were manually added to represent the van der Waals surface of the transmembrane orthosteric pocket of the GHRHR.

The compounds were fitted to the 36 different pharmacophores, and the fit value scoring function was used to rank the compounds from best to worst fitting. A secondary flexible fitting was performed on compounds that had a fit value of more than 3. The resulting unique compounds were ranked according to their score, and the 4326 compounds with a fit value of more than 3.6 were visually inspected for chemical complementarity. Forty-four of these compounds were selected (Supporting Information Figure S1) and purchased for evaluation.

### Molecular Docking

The Alphafold inactive-state specific model of GHRHR from GPCRdb^37^ was used for the docking calculations. Auto Dock Tools were used to prepare the corresponding protein file with atom partial charges assigned, and compounds were docked into the binding site of the receptor using AutoDock Vina. The protein was held rigid during the docking process, while the ligands were allowed to be flexible. Docking simulations were performed using a grid box with dimensions of 20 × 20 × 20 Å and a search space of 10 binding modes, and the exhaustiveness parameter was set to 20.

### Molecular Dynamics Simulations

Molecular Dynamics (MD) simulations of GHRHR in complex with the initial hit compound MK04 and one of the best optimized hit MK04 compounds, MK04–6, were undertaken. All simulations were performed using the GROMACS 2023.1 simulation package^38^ in an NPT ensemble with Periodic Boundary Conditions (PBC). The simulations were performed in triplicates. The systems were embedded into a lipid bilayer composed of phosphatidylcholine (POPC) using the OPM database^39^ and the CHARMM-GUI membrane builder.^40^ The simulated systems consisted of 260 POPC molecules and were solvated using the TIP3P water model in a periodic size box of 100 × 100 × 134 Å^3^. Sodium and chloride ions were added to neutralize the charge of each system with a concentration of NaCl of 0.15 mol/L. The FF19SB force field^41^ was used for the parametrization of the proteins and the POPC lipids, and GAFF2^42^ was used to model the ligands.

Prior to MD simulations, the protein–ligand complexes were subjected to 5,000 steps of energy minimization using the steepest descent algorithm, followed by a standard six-step CHARMM-GUI equilibration protocol. Briefly, the first two equilibration steps were carried out in the NVT ensemble (500 ps for each step with a time step of 1 fs) at a constant temperature of 310 K using the v-rescale thermostat with a coupling constant of 1 ps. The next four steps were performed in the NPT ensemble (125 ps with a time step of 1 fs for the first step and 500 ps with a 2 fs time step for the other three steps) at a constant temperature of 310 K using again the v-rescale thermostat and a constant pressure of 1 bar using the c-rescale barostat with a time constant of 2 ps and compressibility of 4.5 × 10^–5^ bar^–1^ with gradually decreased restraint force constants to various components. These components include harmonic restraints on heavy atoms of the protein, planar restraints to hold the position of lipid head groups of membranes along the *Z*-axis, dihedral restraints to keep fatty acid chain double bonds in the *cis* conformation, and C2 chirality for each lipid molecule. Once equilibrated at constant pressure, 1 μs unbiased MD simulations were performed with the atomic coordinates of each system saved every 100 ps. Covalent bonds with hydrogen atoms were constrained using the LINear Constraint Solver (LINCS) algorithm allowing for a 2 fs time step. Temperature control was kept at 310 K using the Nosé–Hoover thermostat with a coupling constant of 1 ps, while a target pressure of 1 bar was maintained isotropically using the Parinello-Rahman barostat with a time constant of 5 ps and compressibility of 4.5 × 10^–5^ bar^–1^. Long-range electrostatic interactions were treated with the particle-mesh Ewald method with a maximum grid spacing of 1.2 Å. Finally, nonbonded interactions were calculated with a cutoff of 12 Å and a switching distance of 10 Å. The MD trajectories were postprocessed and analyzed using the standard GROMACS tool (gmx_rms) for the calculation of the ligands’ root-mean-square deviation (RMSD) and the protein’s backbone RMSD as a function of time using the starting frame as a reference structure. GetContacts software was used for the calculation of the frequency of the interactions between each ligand and receptor.^43^

### Cell Culture and Transfection

Human embryonic kidney cells stably expressing large T antigen (HEK 293T, catalog no. CRL-3216, RRID:CVCL_0063, from ATCC, Manassas, VA, USA) were cultured in Dulbecco’s Modified Eagle Medium containing Glutamax (DMEM, Life Technologies; Carlsbad, CA, USA) supplemented with 10% (v/v) fetal calf serum (Biochrome; Berlin, Germany) in a humidified incubator at 37 °C with 5% CO_2_. To aid cell attachment, assay plates were precoated with a 1:30 dilution of Matrigel growth factor-reduced basement membrane matrix (Corning; Corning, NY, USA) prior to cell seeding. Cells were transiently transfected with expression vectors using X-tremeGENE HP DNA transfection reagent (Roche; Basel, Switzerland) at a 2:1 ratio.

### cAMP ELISA

Cells were seeded at 2 × 10^5^ cells per well in 24-well tissue culture plates and, 24 h post seeding, were transfected with 0.5 μg/well GHRHR, GLP-1R, or empty vector. Twenty-four hours post-transfection, cells were incubated in the presence/absence of a range of concentrations of GHRH (for dose–response analyses), 1 nM GHRH (a concentration that elicits approximately 30% of the maximal response in this system; Supporting Information Figure S2), or 10 μM test compounds for 1 h at 37 °C (for measurement of agonism), or they were preincubated for 30 min with 10 μM test compounds, 1 μM JV-1–36, or vehicle (Veh) prior to incubation with 10 nM GHRH, 1 nM GLP-1, 10 μM FSK, or Veh for a further 1 h (for measurement of antagonism). Cells were then lysed in lysis buffer (0.1 M HCl supplemented with 0.1% (v/v) Triton X-100) and rocked on a plate rocker for 20 min. Lysates were cleared by centrifugation and diluted in lysis buffer prior to measurement of cAMP (to ensure detection of agonist-stimulated responses within assay limits). Generated cAMP was measured using a cAMP ELISA kit (Enzo Life Sciences Inc., Farmingdale, NY, USA; RRID: AB_2890930) with absorbance at 415 nm being quantified using an iMark microplate reader (Bio-Rad Laboratories; Hercules, CA, USA). Unknown values were interpolated from a standard curve, and calculated cAMP concentrations were adjusted for lysis volumes and dilution factors.

### Crystal Violet Cell Viability Assay

Cells were seeded, transfected, and stimulated as per cAMP ELISA, described above. Instead of addition of lysis buffer, following compound incubations, cells were fixed by incubation with 1% (v/v) glutaraldehyde for 15 min at room temperature. The glutaraldehyde was removed, and cells were stained with 0.1% (w/v) crystal violet for 1 h at room temperature. Wells were washed with ultrapure water until running clear and were left to dry overnight before solubilization of the crystal violet with 1% (w/v) sodium dodecyl sulfate and measurement of absorbance at 595 nm using an iMark microplate reader (Bio-Rad Laboratories).

### Data and Statistical Analysis

All data and statistical analyses were performed using GraphPad Prism, Version 8.0.1 for Windows (GraphPad Software, San Diego, California USA). To reduce unwanted sources of variation (interassay variability), data from each independent experiment were calculated as a ratio of the sum of all data generated in that experiment. For antagonism analyses, to aid in visualization of the magnitude of the effect of the test compounds on agonist-induced responses, data are presented as a percentage (%) of the mean Veh+agonist response. Likewise, for agonism analyses, data are presented as % of the mean agonist response, and for cell viability analyses data are presented as % of the mean of Veh treated samples. For dose response analyses, data are presented as % of matched maximal response and were fitted to sigmoidal dose response curves with a Hill coefficient of unity. Mean pEC_50_ values were calculated and used to determine GHRH potency. For statistical analyses, a one-way ANOVA, followed by Dunnett’s *post hoc* test, was utilized for multiple comparisons or Student’s *t* test for two comparisons, with *p* < 0.05 considered significant.

## Results

### Virtual Screening

For Class B1 GPCRs, recent structures depicting receptors in active or inactive states have facilitated the computational use of models for *in silico* targeting and structure–function studies. In the case of GHRHR, at the initiation of this study, the absence of an experimental structure necessitated the construction of a homology model of the 7TMD region using the structure of the closely related human GCGR in the inactive state. The 7TMD helical regions of the two receptors share a sequence similarity of 40%, suitable for an accurate homology model, given the typically low sequence conservation between GPCRs in this region.^44^ Residues identified to form the 7TMD binding site were Y133^1.43b^, K175^2.60b^, V179^2.64b^, S209^3.36b^, T213^3.40b^, I285^5.39b^, Y342^6.53b^, F345^6.56b^, and L362^7.43b^ and were considered for the structure-based pharmacophore mapping. Although this pharmacophore was based on a homology model, later structures of the active state^7^ and Alphafold model of the inactive state of the GHRHR validated our initial model, as only minor differences were observed in the 7TMD orthosteric cavity (Supporting Information Figure S3).

**Figure 1:**
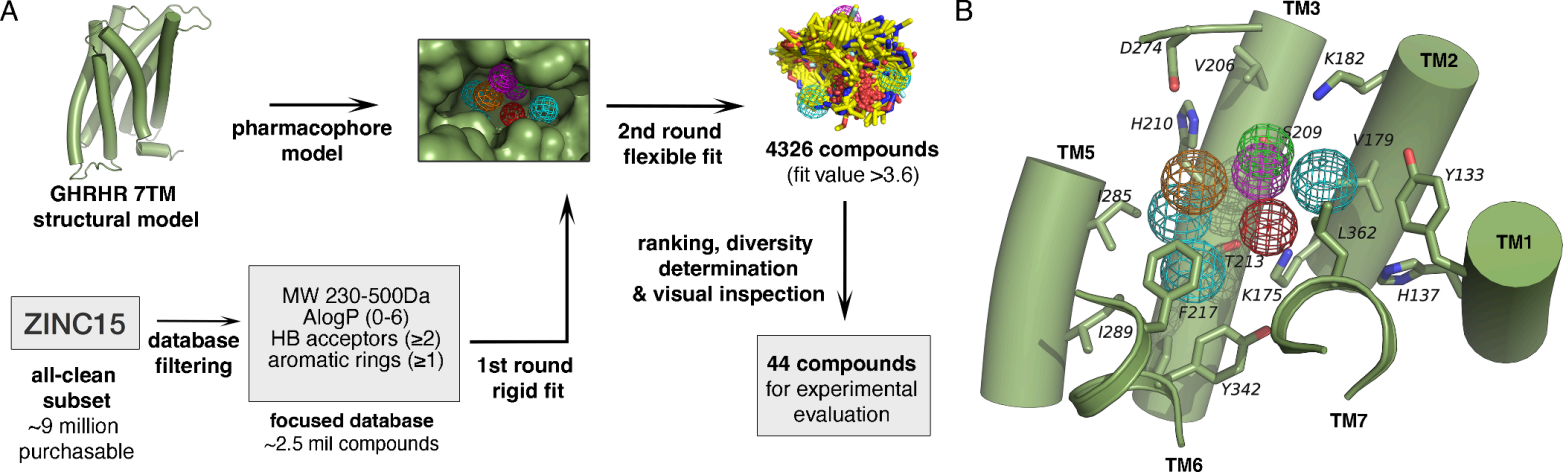
(**A**) GHRHR small-molecule virtual screening procedure. A homology model of GHRHR was constructed and used for the generation of a structure-based pharmacophore model. The all-clean subset from the ZINC15 database was filtered for selected physicochemical properties and initially screened on the pharmacophore model. A second round of screening using a flexible fitting algorithm was performed, followed by ranking based on fit values, diversity determination, and visual inspection resulting in a subset of forty-four compounds for experimental validation. (**B**) The initial structure-based pharmacophore model consisting of a hydrogen bond acceptor (green), a hydrogen bond donor (magenta), an aromatic ring (orange), three hydrophobic features (cyan), and a negative charge (red). Receptor is shown in green.

GHRHR has been targeted in the past by modified peptides but not by small-molecule compounds. To identify potential small-molecule antagonists of GHRHR, we performed a structure-based drug discovery approach on the generated structural homology model (Figure 1A). The pharmacophore used for the virtual screening consisted of one hydrogen bond acceptor (S209^3.36b^), one hydrogen bond donor (S209^3.36b^), one aromatic ring (F345^6.56b^), three hydrophobic features (Y133^1.43b^, V179^2.64b^, T213^3.40b^, I285^5.39b^, Y342^6.53b^, F345^6.56b^, and L362^7.43b^), and one negative charge (K175^2.60b^) (Figure 1B).

After the initial filtering of compounds based on physicochemical properties, the resulting molecules were screened through the pharmacophore models using a rigid fit to obtain a smaller set of potentially active compounds. A flexible fit was applied to this set to refine the final list of compounds, from which the highest scoring (fit value >3.6) was visually inspected for binding site complementarity and individual chemical features, a standard practice for cherry-picking compounds for experimental testing. The selected compounds (MK01-MK44, Supporting Information Figure S1) were purchased and evaluated *in vitro* for their GHRHR activity.

### GHRHR Activity and Hit Expansion

GHRH activation of the GHRHR stimulates downstream activation of Gαs family of G proteins that results in the generation of the second messenger, cAMP. Activity of putative antagonists could therefore be determined using a cAMP ELISA, by exposing cells expressing the GHRHR to the compounds in the presence of GHRH. Cells exogenously expressing human GHRHR were preincubated with 10 μM of each compound prior to stimulation with 10 nM GHRH (a concentration corresponding to approximately 3x the EC_50_ of GHRH measured in this model system, Supporting Information Figure S2).

**Figure 2:**
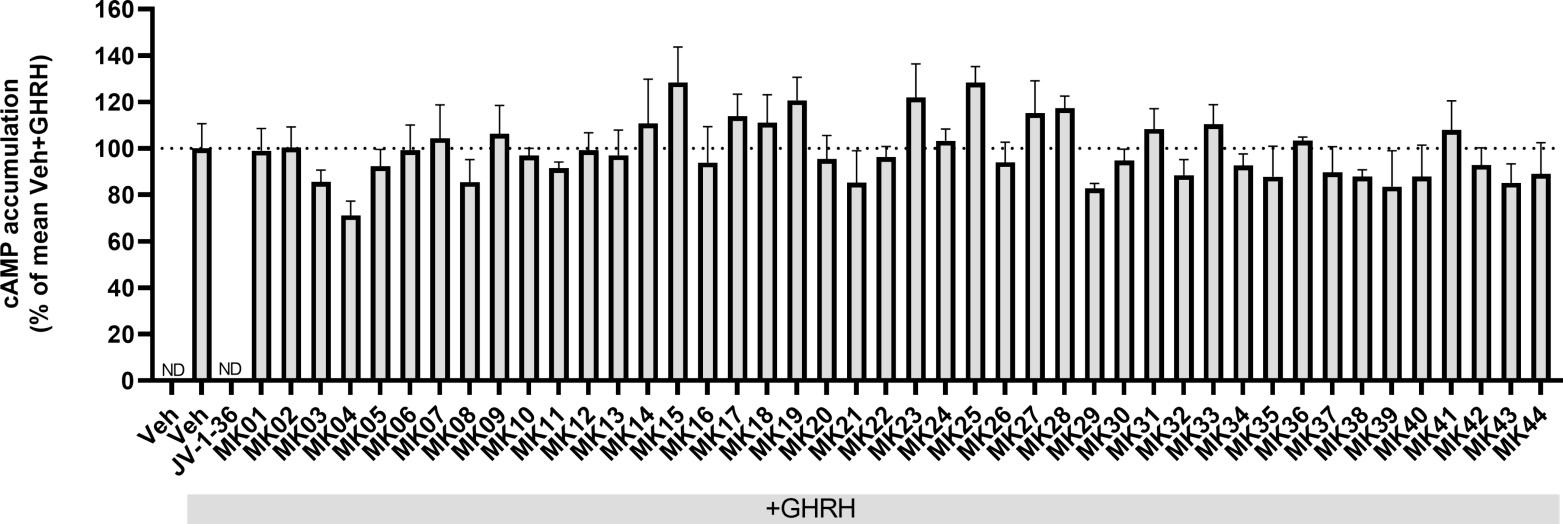
Inhibition of GHRH-stimulated cAMP generation by test compounds MK01 to MK44. HEK 293T cells transiently transfected with GHRHR were preincubated with vehicle, 1 μM JV-1–36, or 10 μM of test compound for 30 min prior to addition of 10 nM GHRH or vehicle for 1 h. Cells were then lysed and generated cAMP measured by ELISA. Data are presented as mean ± SEM from three independent experiments (*N* = 3) and as percentage (%) of the mean Veh+GHRH response. Veh, vehicle; ND, not detectable. No significant difference between Veh+GHRH and test compounds+GHRH (*p* > 0.05, one-way ANOVA followed by Dunnett’s multiple comparisons test).

Some of the test compounds reduced the level of cAMP generated by GHRH stimulation, although these effects were not statistically significant, and the magnitudes of these effects were much less than that seen for the peptide antagonist, JV-1–36, which completely abolished GHRH-induced cAMP generation (Figure 2).

Compound MK04 exhibited the largest decrease (29% inhibition) and was thus used as a starting point for hit expansion. We sought to expand the chemical space around MK04, by selecting and purchasing readily available compounds for evaluation, to perform a structure–activity relationship analysis and dissect the molecular basis of the interaction with the receptor. On this basis, nine molecules were selected (MK04–2-MK04–10, Supporting Information Figure S4), using a Tanimoto chemical fingerprint similarity search on the initial in stock VS library.

**Figure 3:**
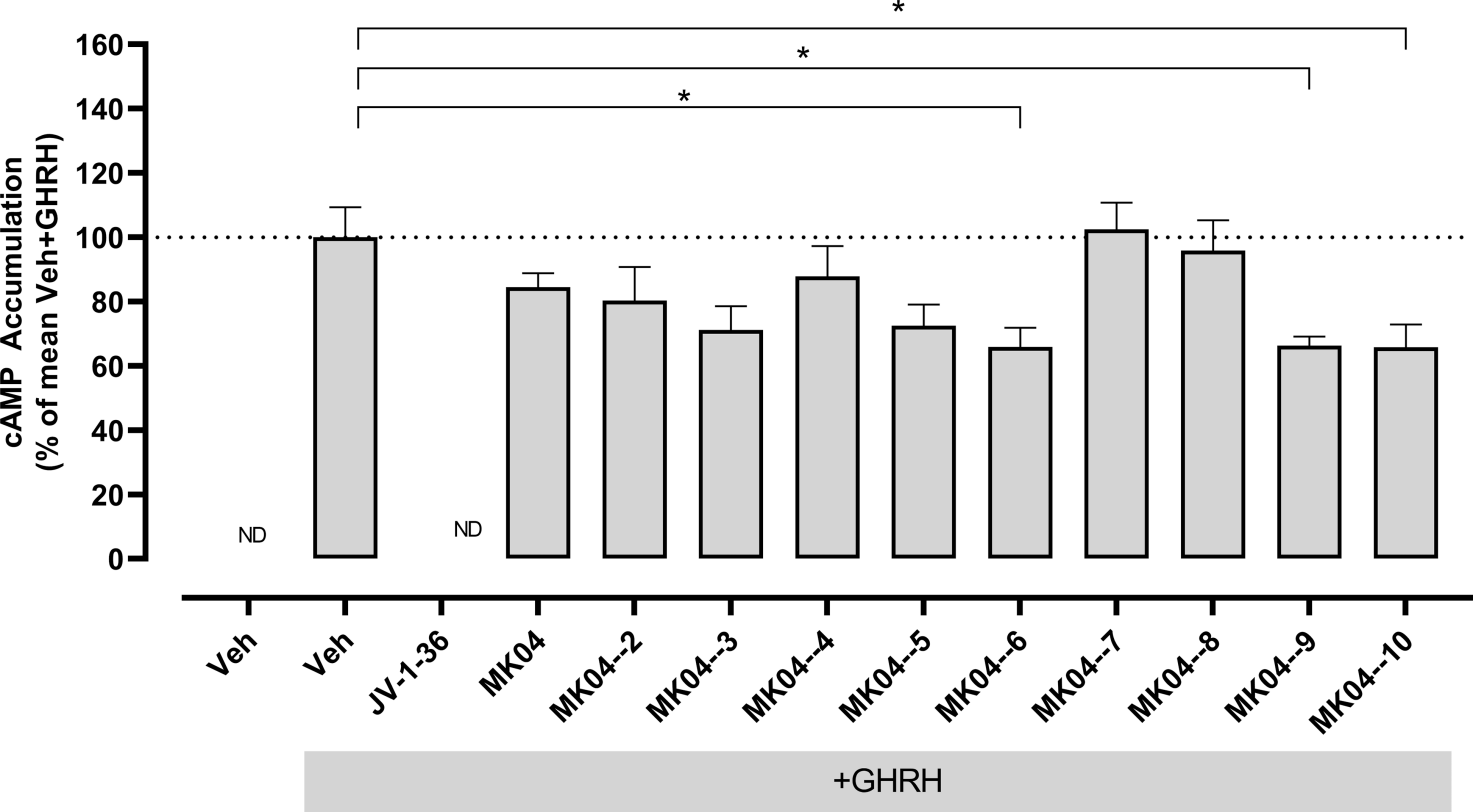
Inhibition of GHRH-stimulated cAMP generation by test compounds MK04–2 to MK04–10. HEK 293T cells transiently transfected with GHRHR were preincubated with vehicle, 1 μM JV-1–36, or 10 μM of test compound for 30 min prior to addition of 10 nM GHRH or vehicle for 1 h. Cells were then lysed and generated cAMP measured by ELISA. Data are presented as mean ± SEM from five independent experiments (*N* = 5) and as percentage (%) of the mean Veh+GHRH response. * *p* < 0.05, one-way ANOVA followed by Dunnett’s multiple comparisons test for comparison with Veh+GHRH. Veh, vehicle; ND, not detectable.

*In vitro* analyses of the effects of these compounds on GHRH-induced cAMP generation revealed that several of the hit expansion series achieved a reduction in the level of GHRH-induced cAMP accumulation (Figure 3).

**Figure 4:**
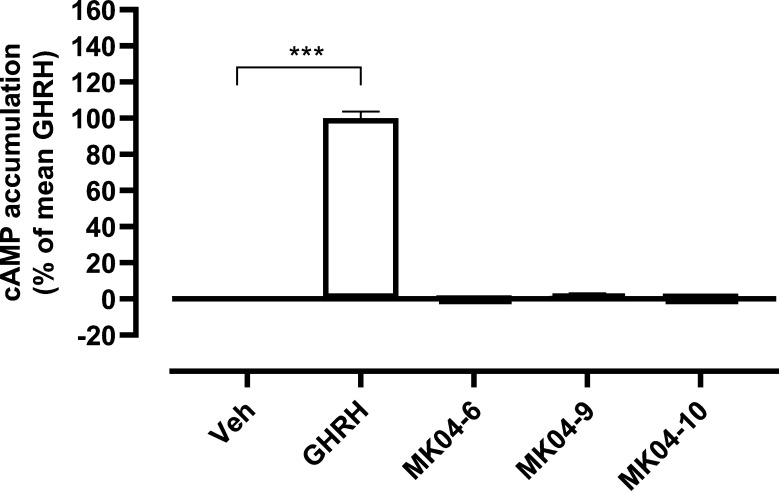
Lack of stimulation of cAMP accumulation by test compounds MK04–6, MK04–9, and MK04–10. HEK 293T cells transiently transfected with GHRHR were incubated with vehicle, 1 nM GHRH, or 10 μM of test compound for 1 h prior to lysis and measurement of generated cAMP by ELISA. Data are presented as mean ± SEM from three independent experiments (*N* = 3) and as percentage (%) of the mean GHRH response. *** *p* < 0.001, one-way ANOVA followed by Dunnett’s multiple comparisons test for comparison with Veh. Veh, vehicle; ND, not detectable.

**Figure 5:**
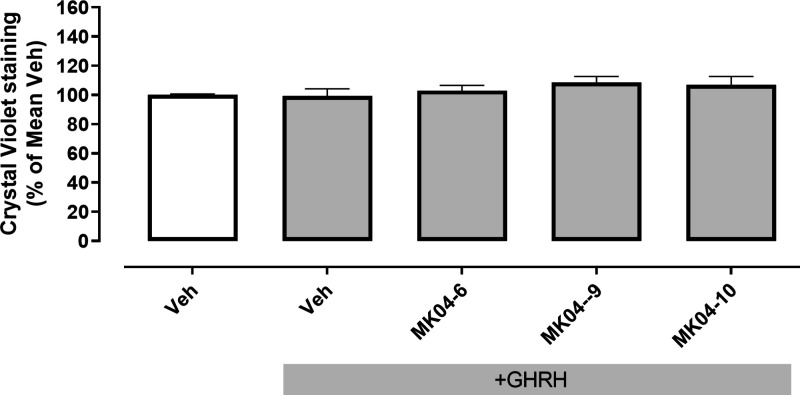
Crystal violet staining of cells expressing GHRHR following incubation with test compounds MK04–6, MK04–9, and MK-10 in combination with GHRH. HEK 293T cells transiently transfected with GHRHR were preincubated with vehicle, 1 μM JV-1–36, or 10 μM of test compound for 30 min before addition of 10 nM GHRH or vehicle for 1 h. Cells were then fixed and stained with crystal violet to quantify viable cell number. Data are presented as mean ± SEM from three independent experiments (*N* = 3) and as percentage (%) of the mean Veh+GHRH response. No significant difference between Veh+GHRH and test compounds+GHRH (*p* > 0.05, one-way ANOVA followed by Dunnett’s multiple comparisons test). Veh, vehicle.

**Figure 6:**
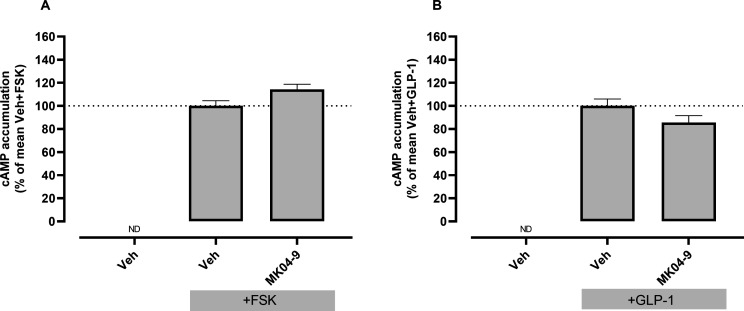
Lack of inhibition of FSK- and GLP-1-induced cAMP accumulation by the test compound MK04–9. HEK 293T cells transiently transfected with empty vector were preincubated with vehicle or 10 μM of test compound for 30 min prior to addition of (**A**) 10 μM FSK or (**B**) 1 nM GLP-1 (concentrations corresponding to approximately 3x EC_50_ of FSK/GLP-1 measured in this model system) or vehicle for 1 h. Cells were then lysed and generated cAMP measured by ELISA. Data are presented as mean ± SEM from (**A**) three (*N* = 3) or (**B**) four (*N* = 4) independent experiments and as percentage (%) of the mean Veh+FSK response or mean Veh+GLP-1 response, respectively. No significant difference between Veh+FSK/GLP-1 and MK04–9+FSK/GLP-1 (*p* > 0.05, Student’s *t* test). Veh, vehicle; ND, not detectable, FSK, forskolin; GLP-1, glucagon-like peptide-1.

Three of these (MK04–6, MK04–9, and MK04–10) achieved a statistically significant reduction of GHRH-induced cAMP accumulation of 34%. Surprisingly, in these experiments the “parent” compound MK04 exhibited a lower degree of inhibition (16%) to that observed previously, possibly reflecting interassay differences or a lack of stability of MK04 when stored in solution. In the absence of GHRH, no cAMP accumulation was elicited by compounds MK04–06, MK04–09, and MK04–10 (Figure 4). Crystal violet staining of cells that had undergone the same treatment regimen confirmed that the observed reduction in cAMP elicited by MK04–6, MK04–9, and MK04–10 was not a result of decreased cell number/viability (Figure 5). Preliminary examination of the selectivity and specificity of these effects was then undertaken using MK04–09 as an example hit compound. No reduction in FSK-induced cAMP accumulation was observed for this compound (Figure 6A), nor was it able to antagonize cAMP accumulation induced by activation of the closely related Class B1 receptor, GLP-1R (Figure 6B), although comparison with a known antagonist of the GLP-1R would be required to confirm this lack of effect.

### Molecular Determinants of Small-Molecule Antagonist Binding

**Figure 7:**
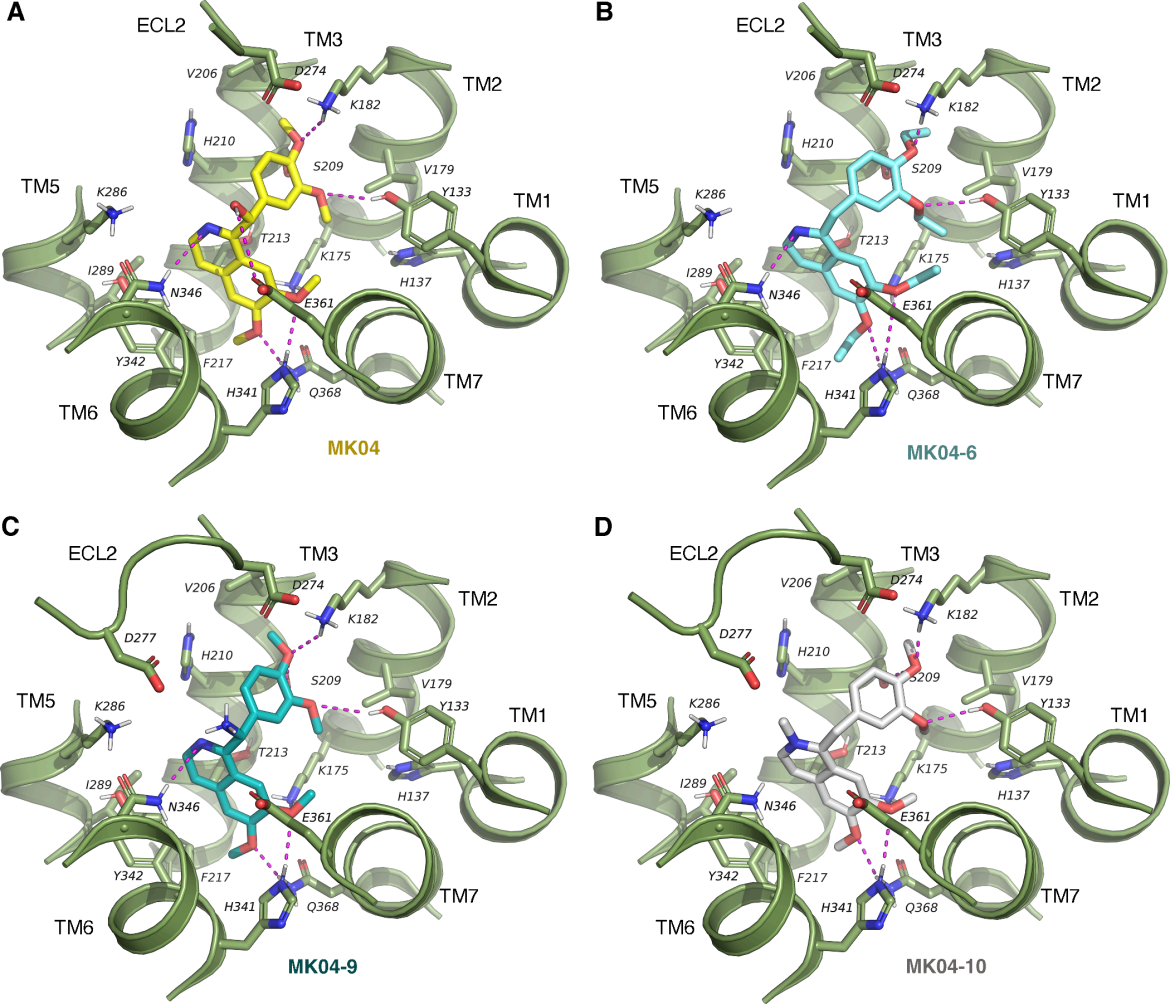
Docking poses of (**A**) MK04 (yellow), (**B**) MK04–6 (cyan), (**C**) MK04–9 (teal), and (**D**) MK04–10 (white) in the 7TMD orthosteric pocket of GHRHR. Receptor and pocket residues are depicted in green, and polar interactions are highlighted in magenta dashed lines.

To understand the molecular basis of successful hits binding to the receptor, we used the newest Alphafold model in the inactive state and performed docking calculations. Specifically, we docked the initial hit compound MK04 and all hit expansion compounds, MK04–2 to MK04–10. Dockings from this series, and especially of MK04–6, MK04–9, and MK04–10, which were found to elicit a significant reduction in GHRH-induced cAMP accumulation, also showed a consistent docking pose with respect to the initial pharmacophore model. MK04 and MK04–9 have a chiral carbon, which was considered during the docking calculations. From the dockings of both R and S counterparts, the MK04 (R) and MK04–9 (R) isomers had more consistent and reasonable interactions with the orthosteric pocket. In terms of the initial hit molecule, MK04 (Figure 7A), the docking mode demonstrates several polar interactions, such as the methoxy oxygens of the phenyl group with K182^2.67b^, S209^3.36b^, and Y133^1.43b^, and the methoxy oxygens of the isoquinoline group with H341^6.52b^ and Q368^7.49b^, all acting as hydrogen bond acceptors. Furthermore, the nitrogen atom from the isoquinoline group may serve as an extra acceptor for the N346^6.57b^ amide group. The characteristic hydroxyl group of MK04 forms a bond with E361^7.42b^. Hydrophobic interactions involve the diaromatic moiety and a methyl group interacting in a hydrophobic region of the pocket, formed by T213^3.40b^, F217^3.44b^, I289^5.43b^, and Y342^6.53b^, and the phenyl ring interacting with H210^3.37b^. For MK04–6, similar interactions are observed (Figure 7B). This molecule has the same core; however, it does not bear the hydroxyl group, “losing” chirality for that reason, and adds the characteristic of ethoxy groups instead of methoxy groups. This may provide extra hydrophobicity, resulting in such interactions in the TM3-TM5-TM6 hydrophobic region, as well as the TM1-TM2 region involving the K175^2.60b^ aliphatic chain, V179^2.64b^ side chain, and the H137^1.47b^ aromatic ring. MK04–9 (Figure 7C), compared to MK04, only has a charged amide group instead of the hydroxyl group, which may interact with D277^ECL2+5^ forming a salt bridge. Besides this interaction, other hydrophobic and polar contacts remain the same as in MK04. MK04–10 (Figure 7D) has a charged nitrogen atom on the isoquinoline group, which may interact with D277^ECL2+5^ through indirect contacts (water molecules) and no hydroxyl group to form a bond with E361^7.42b^. Other hydrophobic and polar contacts remain the same as those in MK04–6. This set of calculations and analysis provides a structure–activity relationship of the MK04 series at the molecular level, as slight differences in the chemical groups result in varying degrees of activity. For example, MK04–2 and MK04–4 show no decrease in GHRH-induced cAMP accumulation, indicating that either an amide at the phenyl group of MK04–2 is incapable of forming a salt bridge with the ECL2 aspartate (D274^ECL2+2^ or D277^ECL2+5^), due to its ortho position on the phenyl ring, or a charged nitrogen on the isoquinoline group which serves as a hydrogen bond acceptor for N346^6.57b^. MK04–7 and MK04–8 were also ineffective in reducing GHRH-induced cAMP accumulation suggesting an sp^2^ geometry at the carbon between phenyl and isoquinoline group results in a noninteracting geometrical conformation of the molecules. MK04–3 which has a methyl group on the isoquinoline nitrogen, maintaining the hydroxyl group, also showed some indication of antagonist activity (although these effects were not statistically significant), which might be attributed to an extra polar interaction with the receptor through water molecules, as no negatively charged residues are located near the charged nitrogen.

To investigate further the binding of representatives of the hit compounds to the receptor, MD simulations were performed on MK04 and MK04–6. MK04–6 does not have a stereocenter and therefore was selected to also assess an active molecule of increased conformational freedom and further validate docking results. We performed three replicas of 1 μs MD simulations using the docking structures of the protein–ligand complexes. Regarding MK04–6, calculation of the ligand and protein 7TMD backbone RMSD with respect to the initial frame showed notably large RMSD values (meanRMSD = 6.92 ± 1.02 Å), indicating the conformational flexibility of the protein–ligand complex from its starting conformation. Supporting Information Figure S5A depicts the RMSD values of MK04-6 and the protein backbone structure during the MD simulations. On the other hand, MK04 demonstrated lower RMSD values (meanRMSD = 5.59 ± 0.60 Å) compared to MK04-6 showing enhanced stability within the binding site (Supporting Information Figure S5B).

In addition, to investigate the structural basis of binding of MK04, MK04–6, and GHRHR, the interactions between the amino acids of the binding site and the ligands were calculated, and their frequency of appearance was extracted. The most important interactions between MK04 and the GHRHR (greater than 40% of the mean frequency of each contact across the three MD simulations) were with residues K175^2.60b^ (44 ± 32%), T213^3.40b^ (48 ± 23%), F217^3.44b^ (66 ± 39%), I289^5.43b^ (45 ± 41%), F338^6.49b^ (65 ± 26%), H341^6.52b^ (63 ± 9%), Y342^6.53b^ (77 ± 32%), E361^7.42b^ (46 ± 25%), G365^7.46b^ (50 ± 43%), and Q368^7.49b^ (51 ± 29%). Hydrophobic interactions, consistent with the docking results, involve the diaromatic moiety formed by T213^3.40b^, F217^3.44b^, I289^5.43b^, and Y342^6.53b^. Moreover, the characteristic hydroxyl group of the ligand forms the same polar interaction with E361^7.42b^. Additional interactions compared with docking results include polar interactions with H341^6.52b^ and Q368^7.49b^ and hydrophobic interactions with the F338^6.49b^ aromatic ring. Finally, it forms an additional hydrogen bond with K175^2.60b^ and hydrophobic interactions with G365^7.46b^. The simulation validated contacts for most of the predicted interactions from the docking predictions, except for the polar interactions with K182^2.67b^, K286^5.40b^, S209^3.36b^, and Y133^1.43b^, maintaining interactions within the pocket.

The most important interactions between MK04–6 and the GHRHR (greater than 40% of the mean frequency of each contact across the three MD simulations) were with residues F217^3.44b^ (60 ± 38%), F338^6.49b^ (64 ± 31%), H341^6.52b^ (76 ± 35%), Y342^6.53b^ (55 ± 31%), G365^7.46b^ (48 ± 22%), and Q368^7.49b^ (57 ± 23%). MK04–6 also interacts with the hydrophobic region of the GHRH pocket formed by F217^3.44b^ and Y342^6.53b^. It also forms the same hydrophobic interactions with the F338^6.49b^ aromatic ring and G365^7.46b^ and similar polar interactions with the ethoxy oxygens of the isoquinoline group with H341^6.52b^ and Q368^7.49b^. Finally, we can also observe that most of the interactions formed in the docking structure are maintained over the course of the MD trajectories.

## Discussion

Many efforts in the past have been made toward identification of small-molecule compounds targeting Class B1 GPCRs. No small-molecule ligands have been reported for GHRHR, although several altered peptides functioning either as antagonists^45^ or as agonists^46^ have been developed. However, peptide ligands can have some limitations with respect to synthesis and administration. In this study we have utilized a structure-based approach to target GHRHR with the aim of identifying compounds that may provide a starting point for further refinement with the potential to produce effective therapeutic small molecules targeting the GHRHR. As GHRHR-targeted peptide antagonists have demonstrated effectiveness in several cancer models, such small-molecule alternatives would likely display similar antimitogenic effects and thus would provide attractive therapeutic options.

Successful compounds resulting from the hit expansion process (MK04–6, MK04–9, and MK04–10), and the initial structure-based pharmacophore hit MK04, all showed a similar profile for decreasing GHRH-induced cAMP accumulation in HEK 293T cells expressing GHRHR. In preliminary analyses, none of the compounds had any effect on viable cell number, confirming that the observed reduction was due to decreased cAMP generation. Furthermore, none elicited any cAMP accumulation in the absence of GHRH, indicating that they act as antagonists rather than as partial agonists. For compound MK04–09, preliminary analyses also suggested there was no reduction in FSK-induced or GLP-1-induced cAMP accumulation, indicating GHRHR selectivity/specificity, although confirmation using a wide panel of GPCRs (and other viable targets) and comparison with known receptor-selective ligands would be required to firmly establish receptor selectivity. The decreases observed for the small-molecule compounds are nowhere near as robust as those achieved by the peptide antagonist JV-1–36, although it is not possible to determine from these single concentration analyses whether this is due to a low potency or efficacy or a combination of the two. Nonetheless, they provide a potential starting point for the further development of more effective compounds.

MK04 and MK04–9 are racemic mixtures. Given the notion that one of the two isomers in each case is the active isomer, we speculate that a greater effect is produced by the MK04–9 active isomer (which our *in silico* data suggest is the R isomer) and masked by the inactive isomer (S isomer). However, this should be experimentally validated. Furthermore, SAR analysis from the MK04 series, all containing an isoquinoline and phenyl group decorated with methoxy and ethoxy groups among others as a core, showed that the isoquinoline nitrogen is important for interaction with N346^6.57b^ and the aromaticity of the group is important for interacting with the TM3, TM5, and TM6 hydrophobic groove, naturally occupied by Tyr^1P^ of GHRH. Replacing methoxy groups with ethoxy also seems to have a slight impact on interactions as hydrophobicity of the molecule is increased and may form further hydrophobic contacts, especially with residues in TM1 and TM2. A charged amide group in the chiral carbon also enhances activity, perhaps through salt bridge formation with the Asp residues in the ECL2 loop, however, not when the charge is present in the quinoline nitrogen. Receptor activation, as highlighted before, is highly dependent on specific contacts with the endogenous peptide,^22^ which among other features, has the ability to produce a widening of the orthosteric pocket and induce a TM6 outward movement and, in part, impair its α-helicity.^7^ Our docking studies suggest that interactions of the MK04 series with TM6, and especially potential hydrogen bonds with N346^6.57b^ and H341^6.52b^, may play a role in inactive state stabilization, which could explain the antagonistic effects. Finally, MD simulations of MK04 and MK04–6 in complex with GHRHR show that the ligands remain stable inside the 7TMD cavity. Moreover, they form hydrophobic and polar interactions in the binding site of the GHRHR suggesting that hydrogen bonds, especially with H341^6.52b^, may be essential for ligand binding. Furthermore, the stability of MK04–6, which does not have a stereocenter and possesses increased conformational freedom, further validates the consistency in docking results. Future confirmation of the *in silico*-derived pharmacophore could be determined by introducing nonsynonymous substitutions into the GHRHR protein or by structural determination of the GHRHR protein in complex with such compounds, e.g., by cryo-EM. This work identifies a novel scaffold for further optimization through medicinal chemistry efforts toward more potent compounds against the GHRHR. Several structural modifications could potentially lead to increased efficacy based on the SAR performed. Specifically, the activity of protonated amide containing MK04–9 would suggest the addition of similar positively charged or hydrogen donor capability groups to reach and interact with D277 of the ECL2, namely, guanidine, primary sulfonamide, triazole, or thiazole. The inhibitory effects of MK04–6 suggest aliphatic ethoxy groups may perform well. Therefore, since the oxygen is crucial for forming hydrogen bonding, substitutions of these ethoxy groups by propoxy, -O-cyclopropyl, or −O-isopropyl would help assess the specifics of hydrophobicity needed for interaction with the pocket. Furthermore, isosteres of the dimethoxyphenyl group, such as trimethoxyphenyl, methylenedioxyphenyl, diethylaminophenyl, or dimethylthiophenyl, could potentially enhance interactions with TM1 and TM2 and specifically Y133^1.43b^, K182^2.67b^, and or D274^ECL2+2^. Such molecules would perhaps enhance interactions with Y133^1.43b^, K182^2.67b^, and or D274^ECL2+2^. Individual or combinations of the above modifications on the 1-phenylisoquinoline scaffold would suffice for an initial hit-to-lead strategy for enhanced activity.

## Conclusions

In this study, we have identified the first small-molecule inhibitors of the GHRHR. A combination of *in vitro* cell-based and *in silico* methods were applied to gain an understanding of their mode of action. It is important to note that the activity of the identified compounds is low, particularly in comparison to existing peptide GHRHR antagonists, which will limit the accuracy of the SAR analyses and conclusions drawn from such. Nonetheless, this study provides a potential starting point for the future design of effective small-molecule GHRHR antagonists.

The current landscape of structurally determined Class B1 receptors with small molecules is mostly composed of allosteric modulators. Indeed, the structures of Class B1 GPCRs that have emerged over the past years have revealed unconventional topologies of small-molecule ligands, such as antagonist MK-0893 in complex with GCGR,^47^ antagonists PF-06372222 and NNC0640 in complex with GLP-1R,^48^ which bind near the intracellular half of the receptor close to TMHs 5–7, restricting the movement of the intracellular tip of TMH6 and antagonist CP-376395, also located in the cytoplasmic half of CRF_1_R.^49^ In contrast, our modeling results point at an inhibitory effect of the MK04 series through the orthosteric 7TMD pocket. We speculate that the decrease in GHRH-induced GHRHR activity is due to a mechanism of blockade of the 7TMD cavity by the efficacious compounds, which fulfill several pharmacophoric characteristics for interacting with the orthosteric pocket as highlighted by the initial screen, dockings, and molecular dynamics simulations. However, an allosteric effect cannot be ruled out, as such mechanisms of small-molecule NAMs or PAMs at Class B1 GPCRs have been thoroughly demonstrated with a vast structural diversity in their action. It is important to appreciate that these *in silico* docking studies are predictive and require future validation of the binding site/interactions. This could be achieved through indirect competitive ligand binding studies and residue mutagenesis analyses or directly through structural analyses of the receptors in complex with the test compounds. These experiments would inform us as to the molecular basis of action of these compounds. Further lead optimization would aim to generate more potent/effective antagonists based on these findings.

## Data Availability

All data and software used in this study are available freely. Ligand structural information is provided in the Supporting Information. The pdb files containing active compound dockings, MD systems’ starting conformations, conformations after 1000 ns, topology files, and parameter files are provided freely at zenodo.org with DOI: 10.5281/zenodo.10914093.

## Supporting Information Available

Details of compound structures (Figures S1 and S4), dose–response analysis of GHRH activation of the GHRHR in this model cell system (Figure S2), structural superposition of homology and alphafold models of GHRHR (Figure S3), and RMSD of MK-04, MK04-06, and GHRHR 7TMD backbone (Figure S5) (PDF)

## Abbreviations

7TMD: seven transmembrane domain
cAMP: cyclic adenosine monophosphate
cryo-EM: cryogenic electron microscopy
DMEM: Dulbecco’s modified Eagle’s medium
DOPE: discrete optimized energy
ECD: extracellular domain
ECL: extracellular loop
FSK: forskolin
GH: growth hormone
GHRH: growth hormone-releasing hormone
GHRHR: growth hormone-releasing hormone receptor
GLP-1: glucagon-like peptide 1
GLP-1R: glucagon-like peptide 1 receptor
GPCR: G protein-coupled receptor
ICL: intracellular loop
IGF-1: insulin-like growth factor 1
MD: molecular dynamics
NAM: Negative allosteric modulator
PAM: Positive allosteric modulator
RMSD: Root mean square deviation
TM: transmembrane helix
VS: Virtual screening
