# Supplementary material for: Identification of Small-Molecule Antagonists Targeting the Growth Hormone Releasing Hormone Receptor (GHRHR)
Source: J Chem Inf Model. 2024 Aug 29;64(18):7056–67. doi: 10.1021/acs.jcim.4c00577 (PMC11423342; doi:10.1021/acs.jcim.4c00577)
Supplement: Supplementary file 1 — ci4c00577_si_001.pdf [file ci4c00577_si_001.pdf]

Identification of small-molecule antagonists targeting the Growth Hormone Releasing Hormone Receptor (GHRHR)

Minos-Timotheos Matsoukas[1,‡,], Tarryn Radomsky[2,3,‡], Vasilis Panagiotopoulos[1], Robin du Preez[2,3,†], Michail Papadourakis[4], Konstantinos Tsianakas[1], Robert P Millar[2,5,6,7], Ross C Anderson[2,3], Georgios A Spyroulias[8], Claire L Newton[2,5,*].*

1 University of West Attica, Department of Biomedical Engineering, Athens, 12243, Greece

2 Centre for Neuroendocrinology, Department of Immunology, Faculty of Health Sciences, University of Pretoria, Private Bag X323, Gezina, Pretoria, 0031, South Africa

3 Department of Physiology, Faculty of Health Sciences, University of Pretoria, Private Bag X323, Gezina, Pretoria, 0031, South Africa

4 Cloudpharm PC, Athens, 15125, Greece

5 Deanery of Biomedical Sciences, University of Edinburgh, Edinburgh, EH8 9JZ, UK

6 Institute of Infectious Diseases and Molecular Medicine, Faculty of Health Sciences, University of Cape Town, Cape Town, 7925, South Africa

7 School of Medicine, University of St Andrews, St Andrews, KY16 9TF, UK

8 University of Patras, School of Health Sciences, Department of Pharmacy, University Campus, Rion, Patras, 26500, Greece

SUPPLEMENTAL INFORMATION

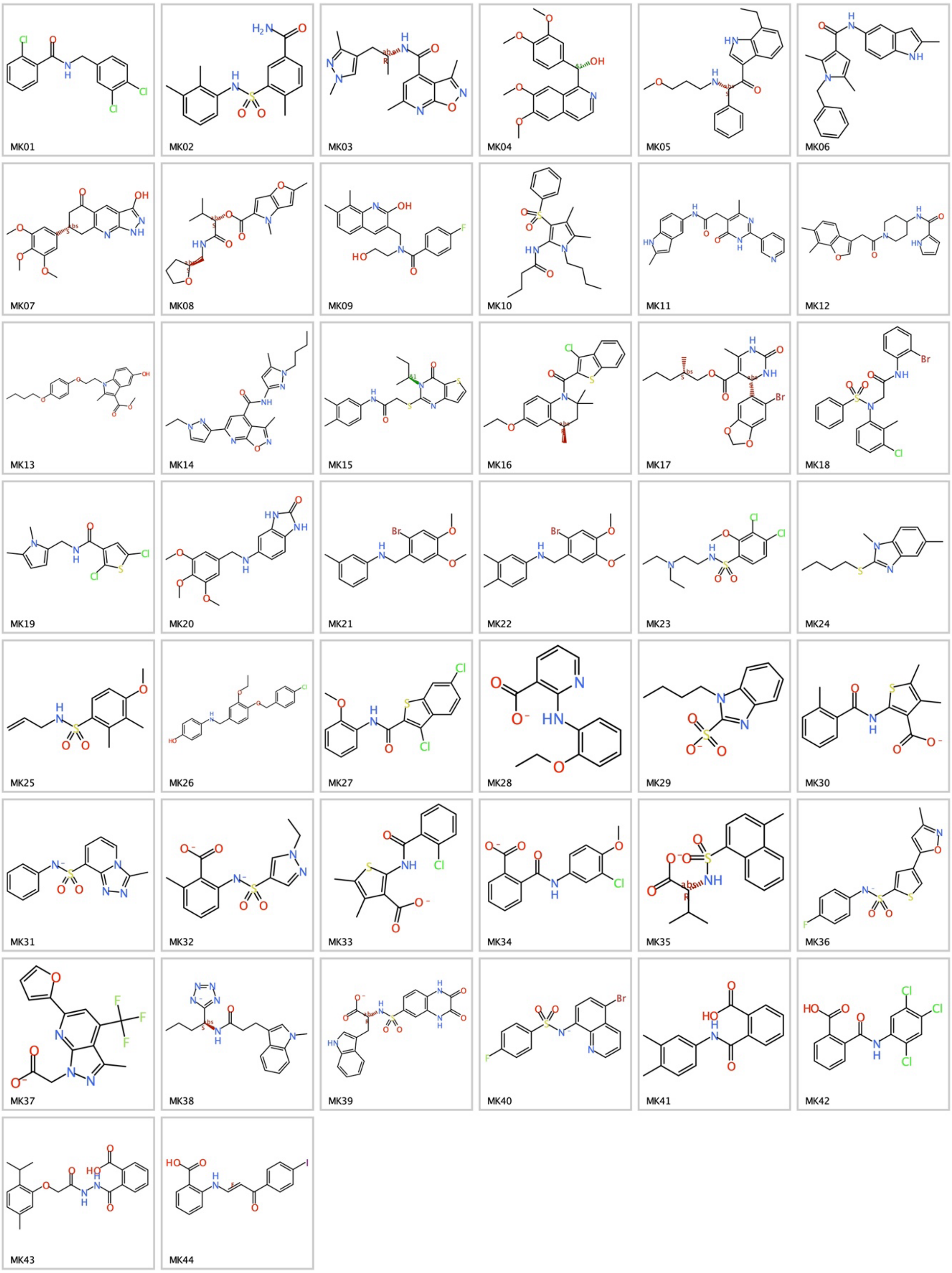

Supplemental Figure S1. Initial compounds selected from the virtual screening for *in vitro* testing.

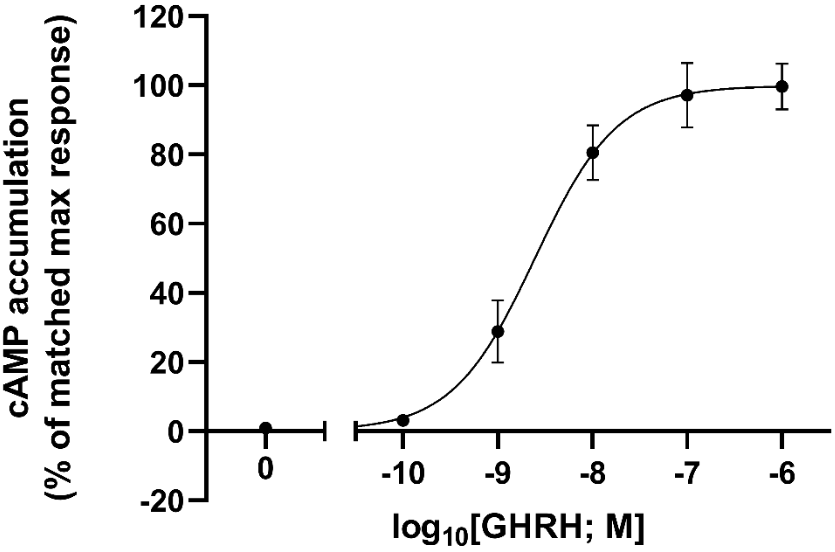

Supplemental Figure S2. cAMP accumulation upon GHRH stimulation of cells transfected with GHRHR. cAMP accumulation was measured by cAMP ELISA in HEK 293T cells transiently transfected with GHRHR following stimulation with a range of GHRH concentrations. Data are presented as mean ± SEM from three independent experiments (N=3) and as % of the matched maximal responses after subtraction of basal stimulation (measured in the absence of ligand; 0). EC_{50} = 3 nM.

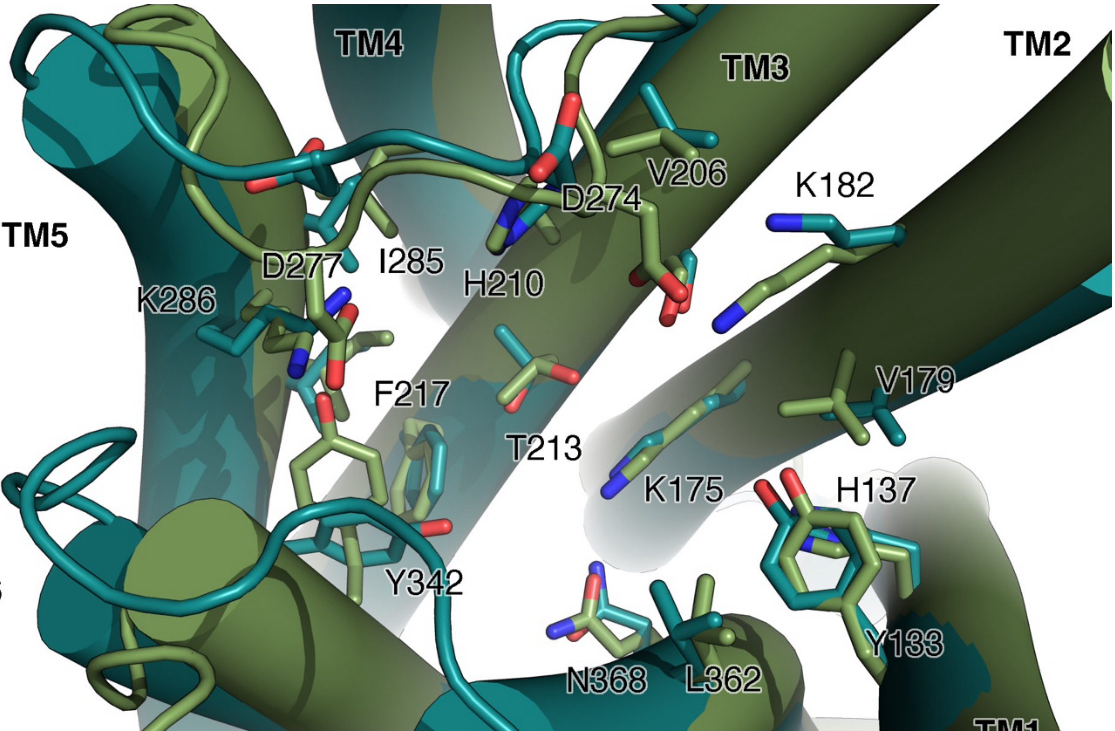

Supplemental Figure S3. Comparison between initial homology model (cyan) of GHRHR and Alphafold 2 model (green).

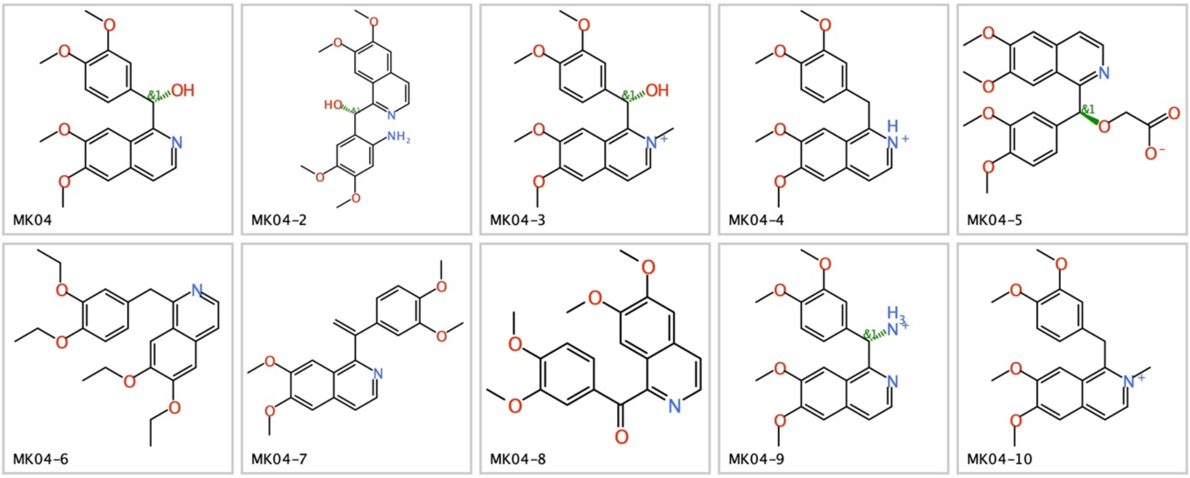

Supplemental Figure S4. Hit expansion series (MK04-2 to MK04-10) selected based on the parent compounds MK04.

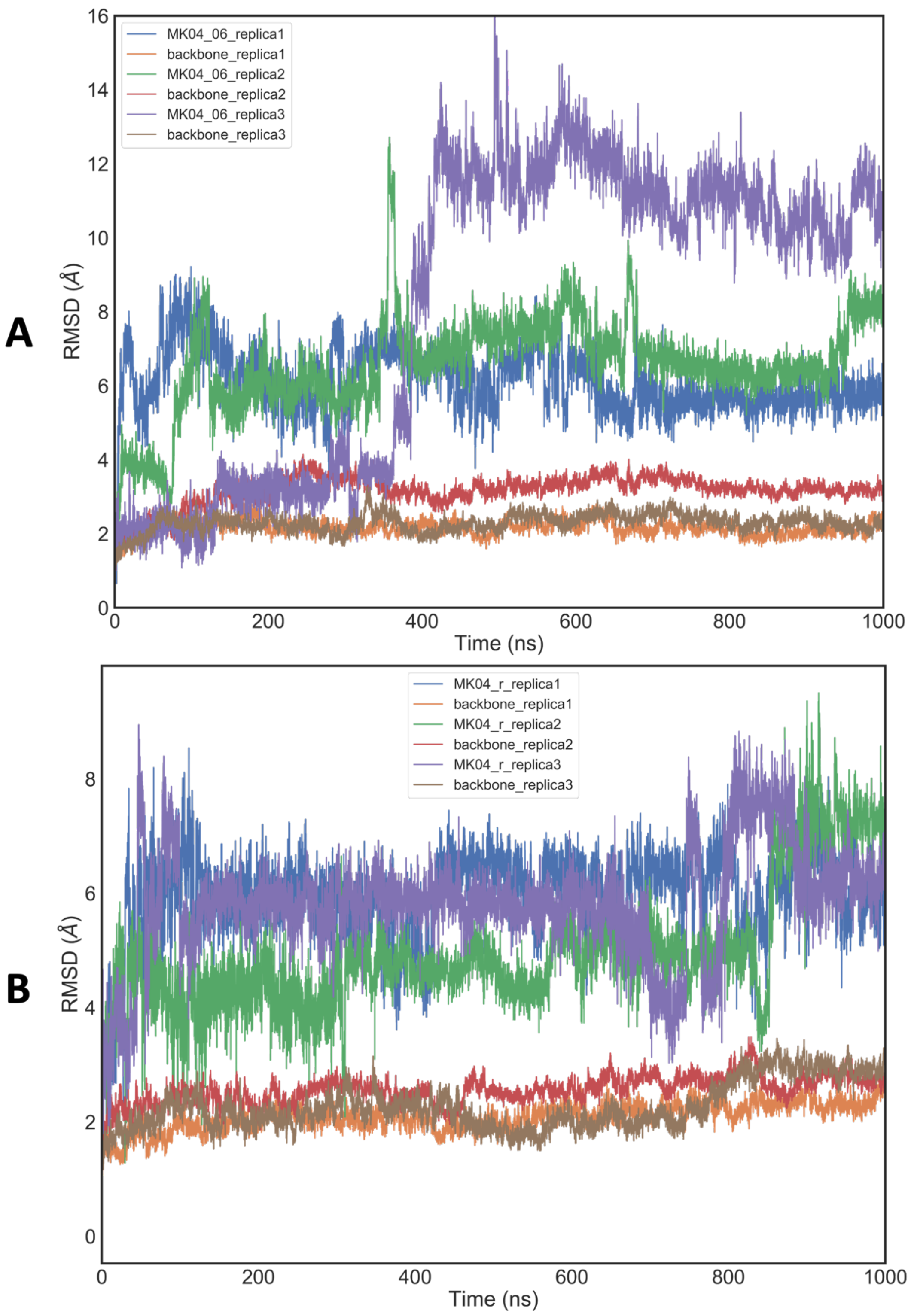

Supplemental Figure S5. RMSD moving average of **A)** MK04_06 (blue, green, and magenta for replicas 1, 2 and 3 respectively) and GHRH 7TMD backbone (orange, red and brown for replicas 1, 2 and 3 respectively) **B)** MK04 (blue, green, and magenta for replica 1, 2 and 3 respectively) and GHRH 7TMD backbone (orange, red and brown for replicas 1, 2 and 3 respectively) as a function of time during the MD simulations.
